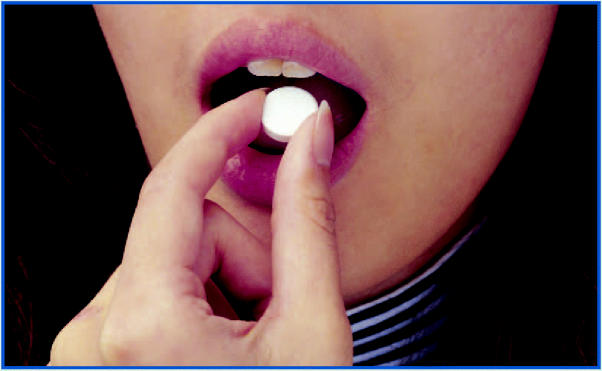# Headliners: Breast Cancer: Regular Aspirin Use May Decrease Breast Cancer Risk

**Published:** 2004-09

**Authors:** Jerry Phelps

Terry MB, Gammon MD, Zhang FF, Tawfik H, Teitelbaum SL, Britton JA, Subbaramaiah K, Dannenberg AJ, Neugut AI. 2004. Association of frequency and duration of aspirin use and hormone receptor status with breast cancer risk. JAMA 291:2433–2440.

Aspirin has been used as a nonprescription pain reliever for more than 100 years, with more than 80 million tablets currently consumed in the United States every day. However, it was not until the 1970s that the mechanism of action was discovered; aspirin was found to inhibit the production of proinflammatory prostaglandins. In the past 20 years, regular aspirin use has been shown to protect against heart disease, stroke, and colorectal cancer. Now NIEHS grantee Marilie Gammon of the University of North Carolina School of Public Health and colleagues report that regular aspirin use may also protect against breast cancer.

Research suggests that inhibition of prostaglandin synthesis may prevent cancer. Cyclooxygenase (COX) is involved in the synthesis of prostaglandins. Aspirin and other nonsteroidal anti-inflammatory drugs (NSAIDs) are known to block the active site of COX and, therefore, inhibit prostaglandin production. Because the final reaction in the synthesis of estrogen depends upon a cytochrome P450 enzyme that is stimulated by prostaglandin E_2_, inhibition of prostaglandin production will also decrease the production of estrogen. Given the importance of estrogen in the development of breast cancer, Gammon and colleagues undertook an epidemiologic study to determine whether there was any association between regular NSAID use and reduced risk of breast cancer.

The team conducted a population-based study of 1,442 women with breast cancer and 1,420 controls. The women were interviewed and asked to report their intake of aspirin, ibuprofen, and acetaminophen. Dose was not considered; instead, the team looked at duration and frequency of use. Regular use was defined as women who took aspirin at least 4 times per week for at least 3 months and initiated use at least 1 year prior to the reference age (age at diagnosis of breast cancer or corresponding age for controls). All exposure information was truncated to 12 months prior to the reference age.

Regular aspirin use was inversely associated with hormone-responsive breast tumors, with the strongest results for women who took 7 or more tablets per week. The results of ibuprofen use were generally weaker. There was no association with use of acetaminophen, which does not inhibit prostaglandin synthesis.

This study adds to the growing body of data that supports the regular use of aspirin as an effective chemopreventive agent for hormone-responsive breast cancer tumors. This effect most likely occurs through the inhibition of prostaglandin and subsequent inhibition of estrogen biosynthesis. However, the reduced risk must be confirmed before clinicians can make definite recommendations to women at risk for breast cancer. **–Jerry Phelps**

## Figures and Tables

**Figure:**